# Supplementary material for: Single-cell multi-omic analysis of the vestibular schwannoma ecosystem uncovers a nerve injury-like state
Source: Nat Commun. 2024 Jan 12;15:478. doi: 10.1038/s41467-023-42762-w (PMC10786875; doi:10.1038/s41467-023-42762-w)
Supplement: Supplementary file 1 — Supplementary Information [file 41467_2023_42762_MOESM1_ESM.pdf]

<u>Supplementary Information</u>

Single-cell multi-omic analysis of the vestibular schwannoma ecosystem uncovers a nerve injury-like state

Thomas F. Barrett[1†], Bhuvic Patel[2†], Saad M. Khan[3,4Ψ], Riley D.Z. Mullins[1,5Ψ], Aldrin K.Y. Yim[5], Sangami Pugazenthi[2], Tatenda Mahlokozera[2], Gregory J. Zipfel[2,6], Jacques A. Herzog[1,6], Michael R. Chicoine[7], Cameron C. Wick[1,6], Nedim Durakovic[1,6], Joshua W. Osbun[2], Matthew Shew[1,6], Alex D. Sweeney[8], Akash J. Patel[8,9,10], Craig A. Buchman[1,6], Allegra A. Petti[3,4*], Sidharth V. Puram[1,5,11*], Albert H. Kim[2,5,6*]

†ΨThese authors contributed equally to this work.

*These authors jointly supervised the work.

1Department of Otolaryngology-Head and Neck Surgery, Washington University School of Medicine, St. Louis, MO, USA.

2Department of Neurological Surgery, Washington University School of Medicine, St. Louis, MO, USA.

3Department of Neurosurgery, Massachusetts General Hospital and Harvard Medical School, Boston, MA, USA.

4Brain Tumor Immunology and Immunotherapy Program, Department of Neurosurgery, Massachusetts General Hospital, Harvard Medical School, Boston, MA

5Department of Genetics, Washington University School of Medicine, St. Louis, MO, USA.

6Brain Tumor Center, Washington University School of Medicine/Siteman Cancer Center, St. Louis, MO, USA

7Department of Neurological Surgery, University of Missouri School of Medicine, Columbia, MA, USA.

8Department of Otolaryngology-Head and Neck Surgery, Baylor College of Medicine, Houston, TX, USA.

9Department of Neurosurgery, Baylor College of Medicine, Houston, TX, USA.

10Jan and Dan Duncan Neurological Research Institute, Texas Children's Hospital, Houston, TX, USA.

11Siteman Cancer Center, Washington University in St. Louis, St. Louis, MO, USA

Supplementary Figure 1. scRNA-seq data integration and VS TME classification by peripheral nerve signatures

Supplementary Figure 2. scATAC-seq quality control and cell type assignment.

Supplementary Figure 3. Supplementary VS-SC analysis

Supplementary Figure 4. Classification of cell states in stromal and immune cell populations.

Supplementary Figure 5. Classification of deconvolved bulk RNA expression data.

Supplementary Figure 6. Supplemental scATAC-seq and ligand-receptor analyses.

Supplementary Figure 7. Ligand-receptor networks and characterization of HSC cell lines.

Supplementary Table 1. scRNA-seq Patient Characteristics

Supplementary Table 2. NF2 mutations derived from WES analysis.

Supplementary Table 3. Number of cells of each cell type with/without chromosome 22q loss from all samples predicted to have chr22q loss by inferCNV and/or WES (SCH1, SCH2, SCH13, SCH20).

Supplementary Table 4. Number of cells of each Schwann cell subtype with/without chromosome 22q loss from all scRNA samples.

Supplementary Table 5. Bulk RNA-seq patient characteristics

Supplementary Table 6. CellBender Parameters

Supplementary Figure 1

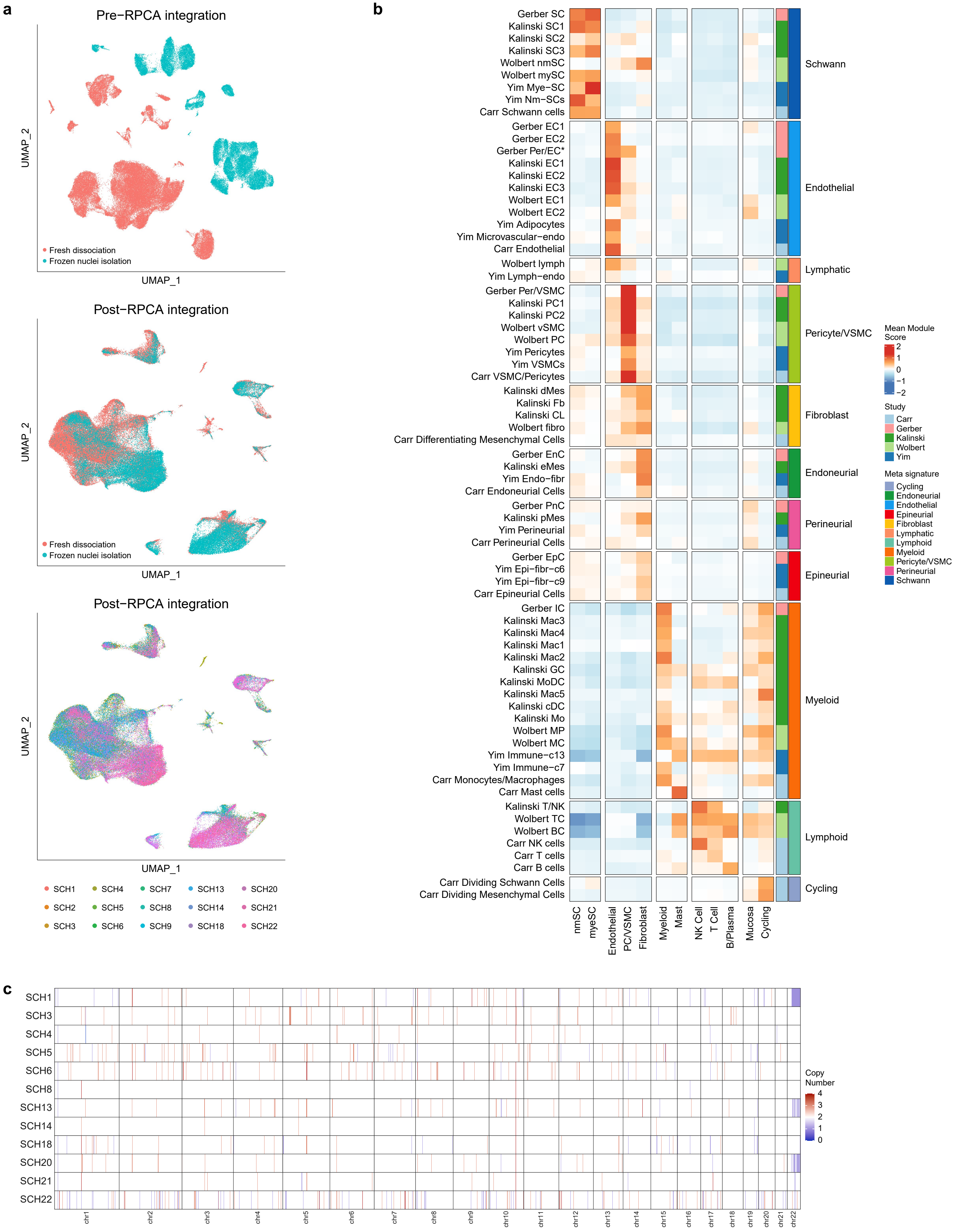

Supplementary Figure 1. scRNA-seq data integration and VS TME classification by peripheral nerve signatures

a. UMAP plot of VS scRNA-seq dataset of embeddings pre-RPCA integration (top), post-integration (middle), and colored by individual sample. Batch effects associated with fresh tissue dissociation and nuclei isolation are overcome.
b. Heatmap showing average mouse peripheral nerve cell-type signature score (rows) for each VS cell-type cluster (columns).
c. Heatmap showing segmental copy number alterations detected by whole exome sequencing of tumors.

Supplementary Figure 2

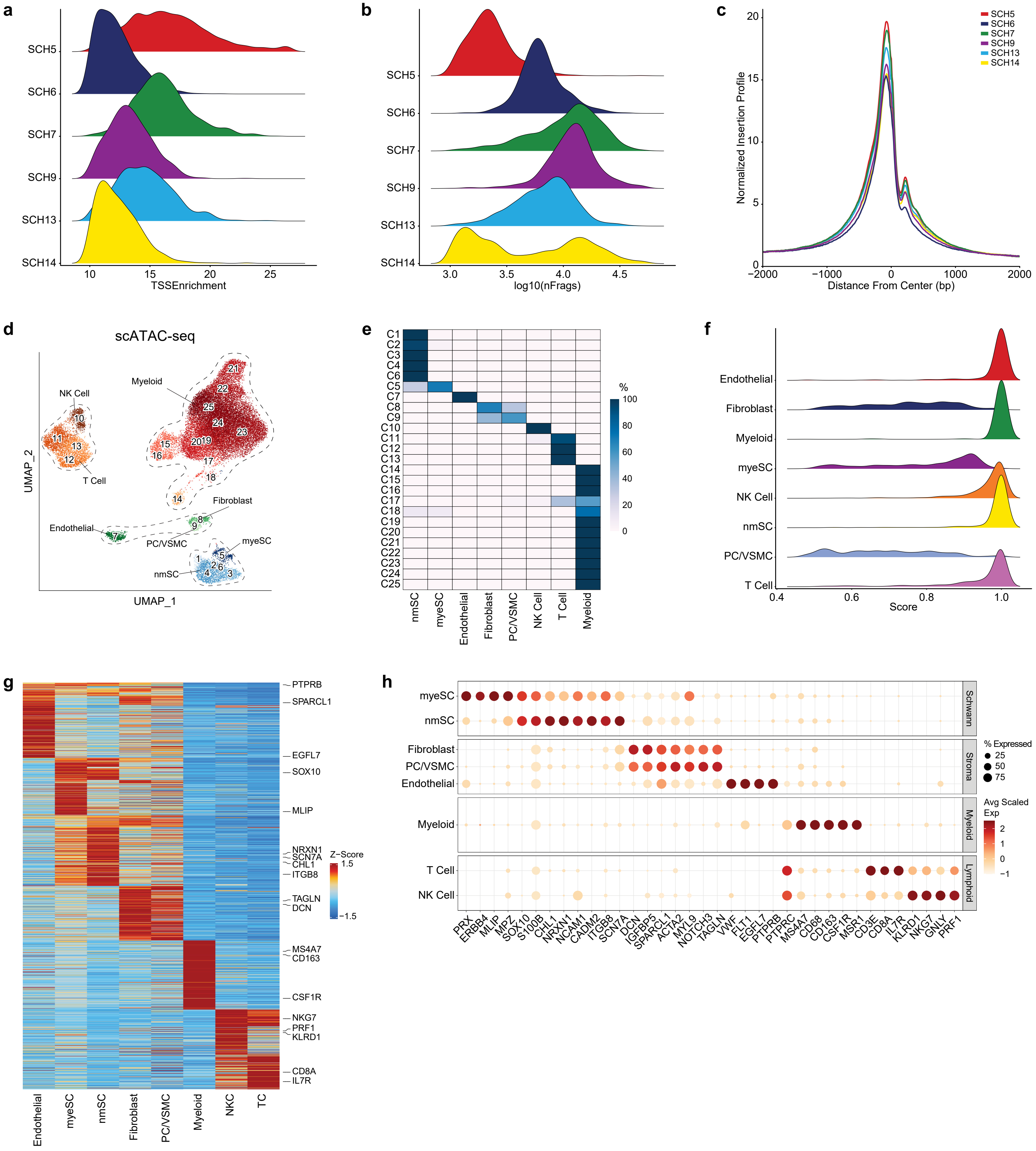

Supplementary Figure 2. scATAC-seq quality control and cell type assignment.

a. Ridgeplot showing distribution of TSS Enrichment for each tumor after preprocessing (removal of cells TSS Enrichment < 10, number of fragments < 1000, and doublet removal as detailed in text).
b. Ridgeplot showing distribution of number of fragments per cell for each tumor after preprocessing.
c. TSS enrichment profile for each sample shows clear peak in center with smaller shoulder peak to the right, consistent with a well-positioned +1 nucleosome and good quality ATAC-seq data.
d. "Over-clustering" of scATAC-seq data at high resolution identifies 25 cell clusters which were initially labeled using inferred expression from gene accessibility of marker genes of various cell types. NK, natural killer cells; VSMC, vascular smooth muscle cells; nmSC, non-myelinating Schwann cells; myeSC, myelinating Schwann cells.
e. Confusion matrix generated after unconstrained linkage of cells from the scRNA-seq dataset with cells in the scATAC-seq dataset. scATAC-seq cells in most clusters where overwhelmingly linked with just one type of scRNA-seq cell. scATAC-seq clusters were ultimately labeled with the identity of the scRNA-seq cell type that most cells in the cluster were linked to.
f. Ridgeplot displaying distribution of score assigned to each scATAC-seq to scRNA-seq cell linkage by ArchR.
g. Heatmap of marker genes for each cell cluster identified from inferred gene expression based on chromatin accessibility.
h. Dot plot of expression levels of characteristic genes described in the literature for each VS cell subpopulation derived from expression data of scRNA-seq cells linked to scATAC-seq cells in each cluster.

Supplementary Figure 3

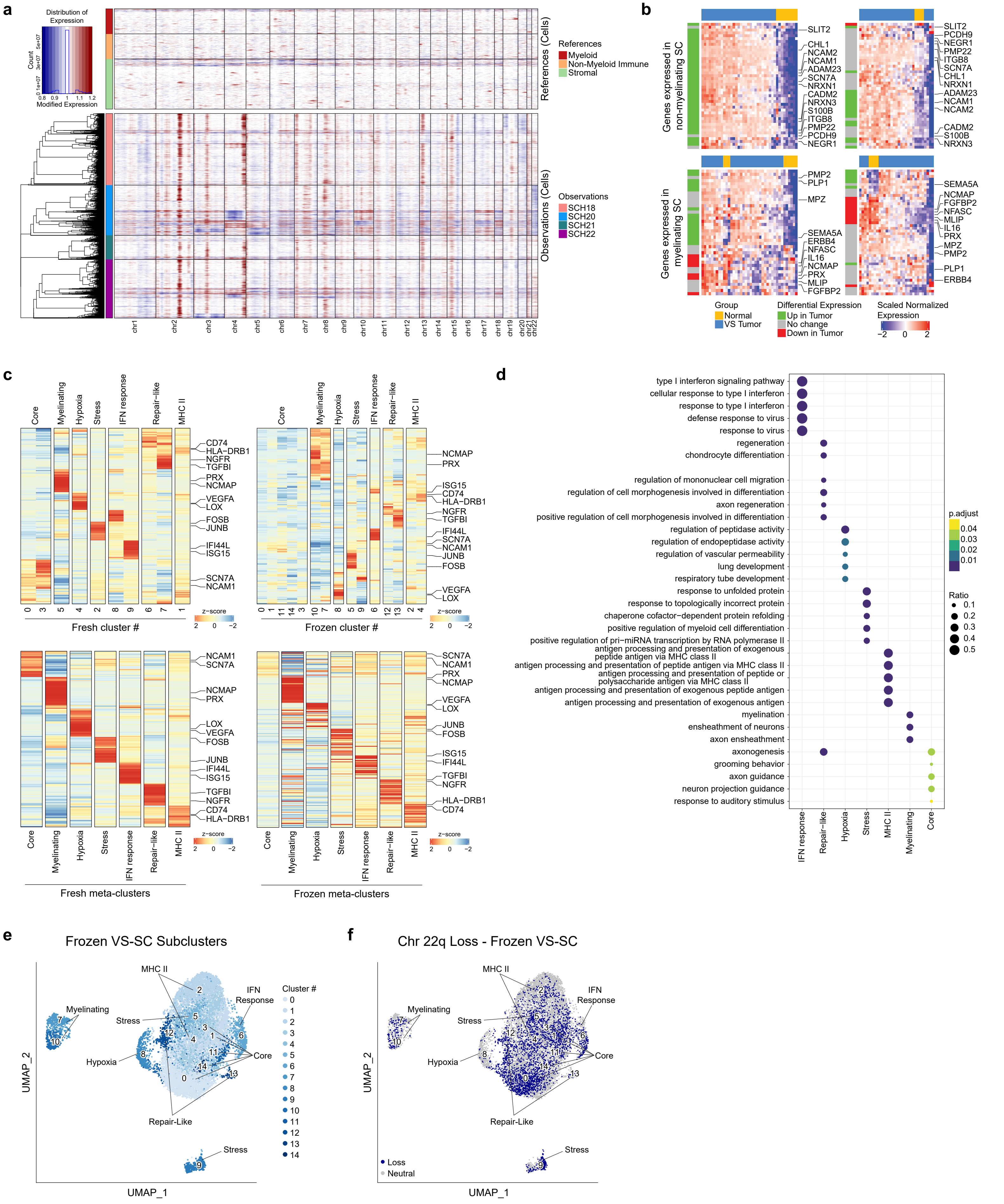

Supplementary Figure 3. Supplementary VS-SC analysis

a. InferCNV residual gene expression heatmap of VS-SC from frozen nuclei samples showing decreased expression of genes on chromosome 22q (chr22q), indicative of chr22q loss, in VS-SC from one tumor (SCH20). See also Fig. 2a for a heatmap of VS-SC from fresh samples that were analyzed independently and identically.
b. Heatmaps comparing expression of top 50 differentially expressed genes (DEGs) in VS-nmSC (top) and VS-myeSC (bottom) to expression observed in microarray data of normal nerve and VS tumors (left, Torres-Martin et al. (GSE39645); right, Zhao et al. (GSE108524); see also Fig. 2c).
c. Heatmaps showing Seurat clustering results for VS-SC subclusters (top) with hierarchical clustering of top 30 DEGs of each cluster. Bottom heatmap shows final cluster-type labels and expression of cluster-defining genes. VS-SC from fresh dissociation (left) and frozen nuclei (right) samples were analyzed independently and identically. There is strong correlation in gene expression and cluster assignment between both groups of samples.
d. Dot plot displaying results from GO BP enrichment analysis for top 25 DEGs from each VS-SC subtype.
e. UMAP representation of VS-SC from the frozen nuclei dataset with meta-clusters labeled. See also Fig. 2e.
f. UMAP plot of frozen nuclei sample VS-SC highlighting cells with inferred chr22q loss. Cells with chr22q loss do not form a discrete cluster but instead cluster with cells without chr22q loss that share the same metaprogram. See also Fig. 2f.

Supplementary Figure 4

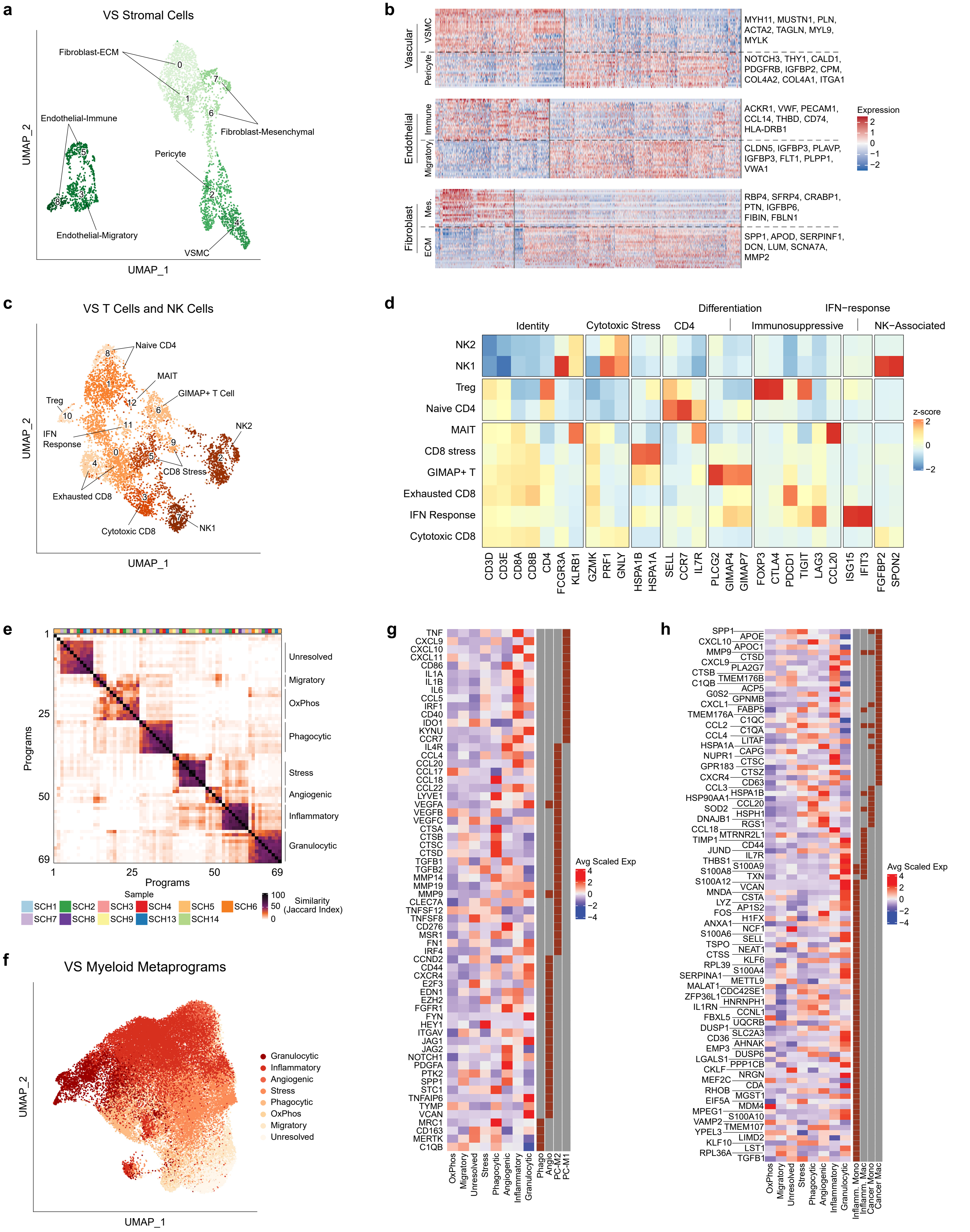

Supplementary Figure 4. Classification of cell states in stromal and immune cell populations.

a. UMAP plot displaying stroma cell meta-cluster labels.
b. Heatmap showing representative gene expression for each stromal cell meta-cluster.
c. UMAP displaying NK and T cell meta-cluster labels.
d. Heatmap showing average expression across meta-clusters of known marker genes for NK and T cell phenotypes.
e. Heatmap displaying pairwise similarities between myeloid-cell programs, identified via NMF. Annotations on right designate metaprogram labels.
f. Myeloid cells were scored for each metaprogram identified in Fig. S4e and assigned to the metaprogram for which they scored highest.
g. Heatmap showing expression of cancer associated macrophage markers, as defined in a pan-cancer (PC) scRNA-seq analysis of tumor infiltrating macrophages[41].
h. Heatmap showing expression of monocyte/macrophage markers expressed in the setting of inflammation and cancer, as defined in pan-tissue scRNA-seq analysis of myeloid cells[43].

Supplementary Figure 5

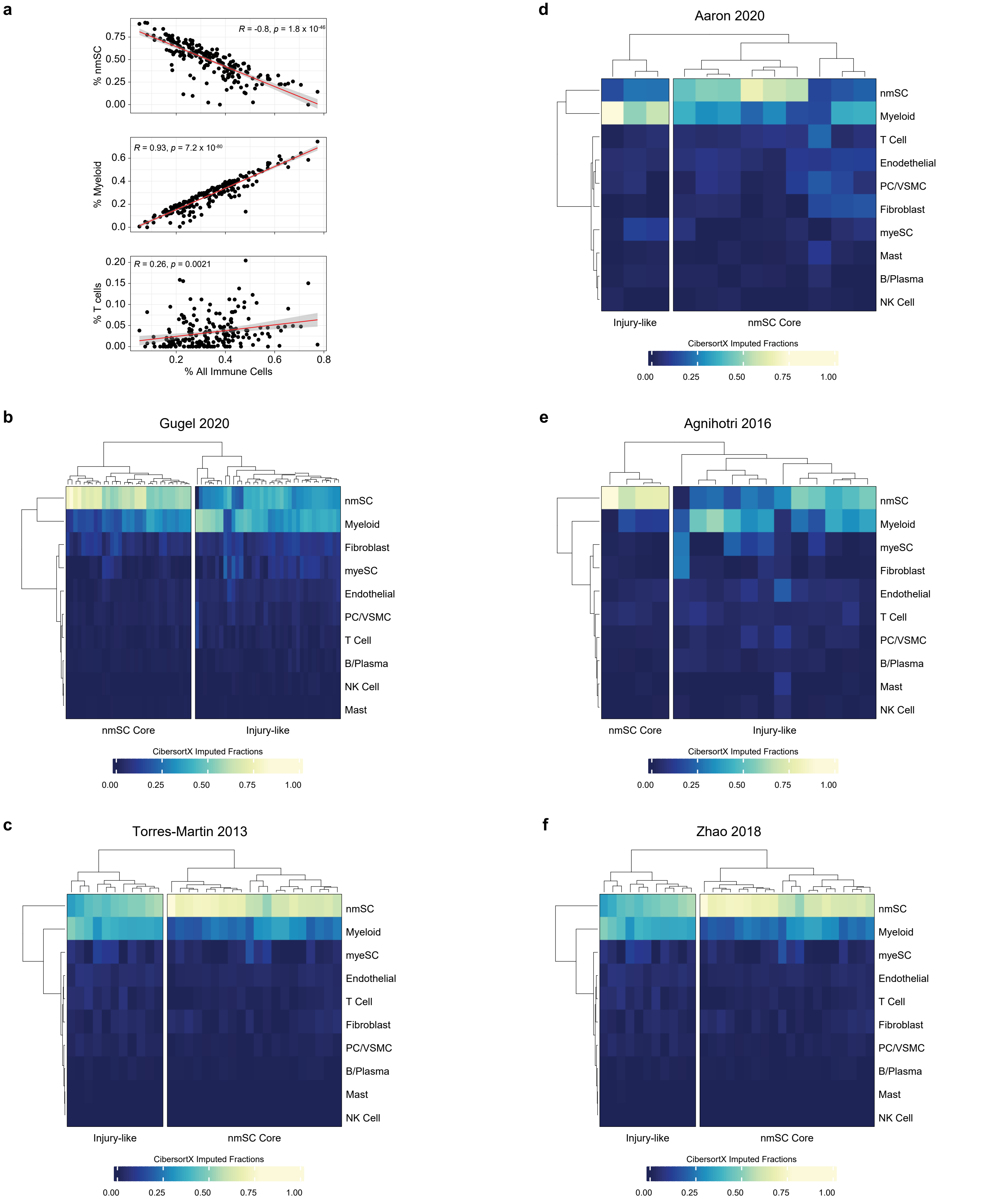

Supplementary Figure 5. Classification of deconvolved bulk RNA expression data.

a. Correlation of imputed fractions of myeloid (top), T cell (middle), and nmSC (cells) with percentage of all immune cells in each deconvolved tumor sample.

b.-f. Heatmaps displaying imputed cell fractions from CIBERSORTx deconvolution. VS tumors are classified into Injury-like and nmSC Core categories using hierarchical clustering of imputed cell fractions. PC/VSMC, pericyte/vascular smooth muscle cells; myeSC, myelinating Schwann cell; nmSC, non-myelinating Schwann cell.

Supplementary Figure 6

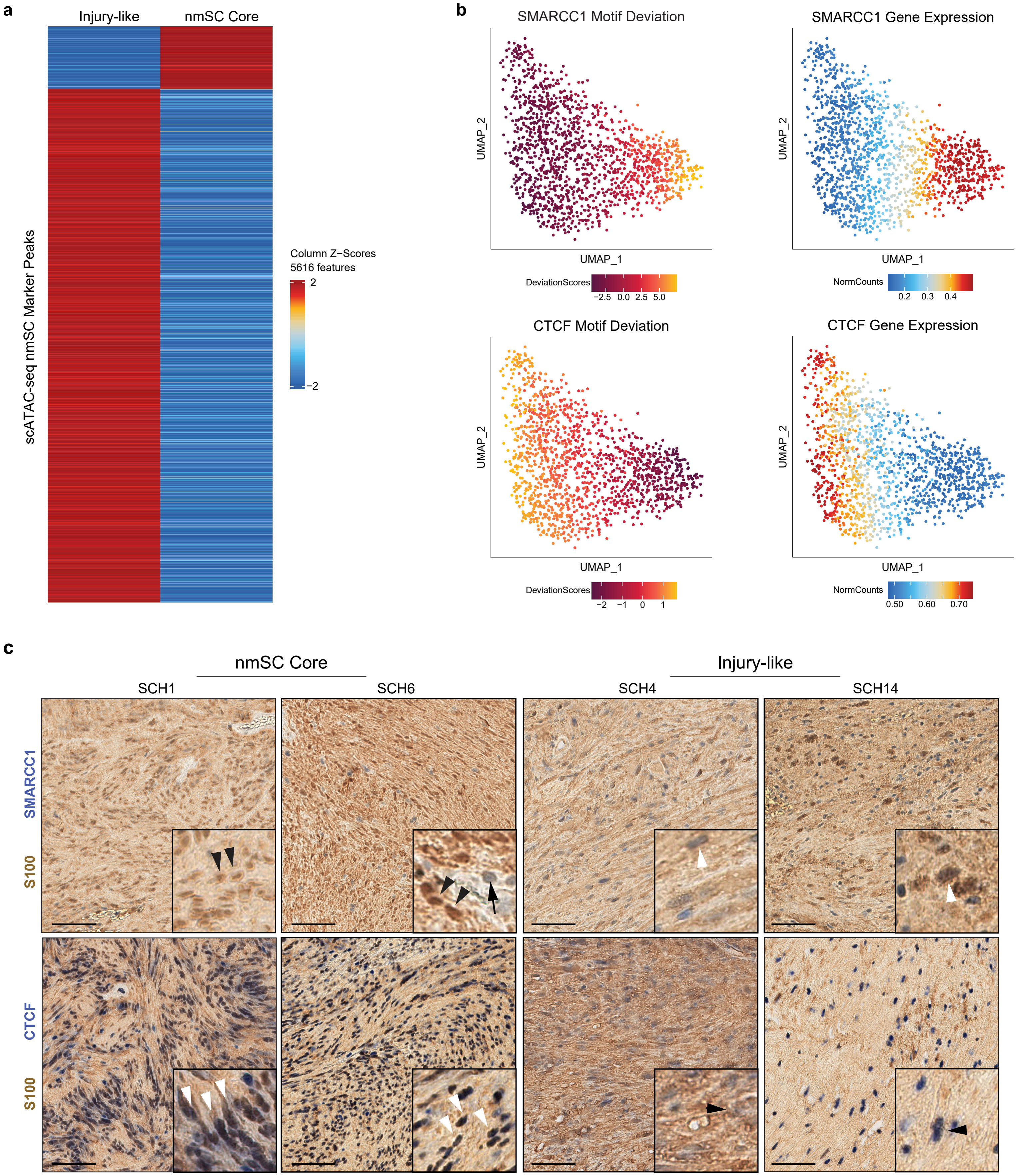

Supplementary Figure 6. Supplemental scATAC-seq and ligand-receptor analyses.

a. Heatmap showing differentially accessible peaks (DAPs) identified 5616 statistically significant marker peaks with Log2FC $\geq$ 2 differentiating VS-SC in Injury-like and nmSC Core tumors.

b. UMAP of Motif Deviation and Gene Expression of select genes specific to nmSC Core and Injury-like VS-SC.

c. Double-stain IHC. Top: There is enrichment of S100+/SMARCC1+ cells (white arrowhead) in Injury-like tumors, whereas S100+/SMARCC1- cells (black arrowhead) and S100-/SMARCC1+ cells (black arrow) are more abundant in nmSC Core tumors. Bottom: There is enrichment in staining intensity and cell density of S100+/CTCF+ cells in nmSC Core tumors. Double positive cells are present, though staining is less intense, in Injury-like VS (black arrowhead). Scale bars = 50 μm.

Supplementary Figure 7

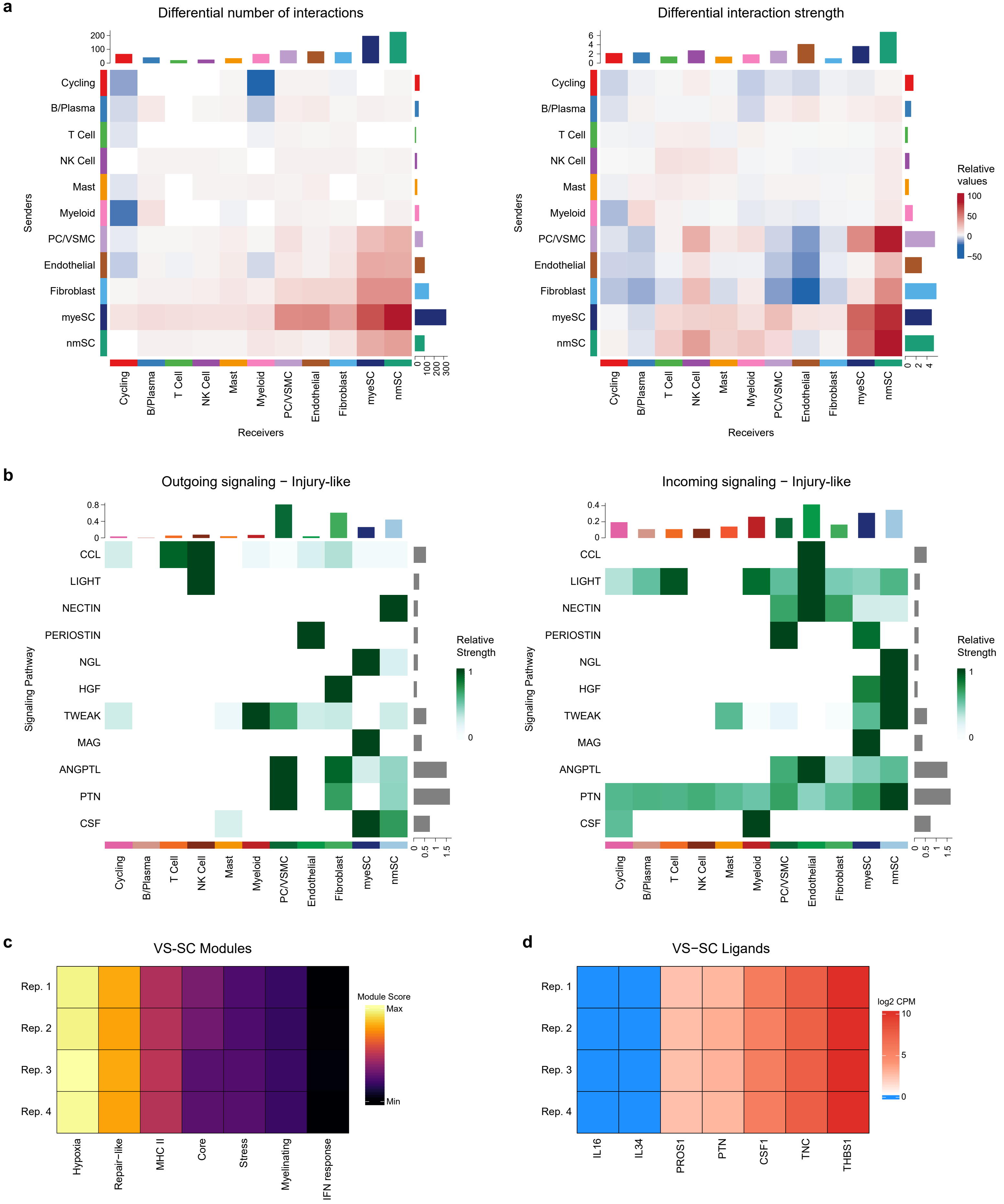

Supplementary Figure 7. Ligand-receptor networks and characterization of HSC cell lines.

a. Heatmap displaying the relative interaction strength of signaling pathways enriched in Injury-like VS. Top bar plots show summative contribution of individual cell types. Side bar plots show summative contribution of a given pathway to the inferred communication network.

b. Heatmap showing differential number of interactions (left) and interaction strength (right), displayed as Injury-like VS relative to nmSC Core VS. The top bar plot represents the sum of column of values displayed (*i.e.*, incoming signals). The right bar plot represents the sum of row of values (*i.e.*, outgoing signals). Red and blue colors in the color scale represent increased and decreased signaling, respectively, in Injury-like VS nmSC Core tumors. PC/VSMC, pericyte/vascular smooth muscle cells; myeSC, myelinating Schwann cell; nmSC, non-myelinating Schwann cell.

c. Bulk RNA-sequencing gene expression profiles obtained from two technical and two biological replicates for the HSC cell line. Scoring HSC bulk expression with the VS-SC meta-cluster gene signatures suggests that the HSC cells most resemble the Repair-like and MHC II VS-SC programs found in Injury-like VS, and less so the Core VS-SC program found in nmSC core VS.

d. Heatmap showing log-transformed expression of candidate VS-SC ligands in HSC cells based on bulk RNA-sequencing.

Supplementary Table 1. scRNA-seq Patient Characteristics

Subject	Technique	scATAC	Sex	Size	Consi	Subjective_He	Previous_Treatment	Dysequilibrium	Trigem	WRS	PT50	PT100	PT2000	PTA	AAO-HNS	Resection	Adherent	FN_Stim	Postop_Fl	Postop_Recurrence
SCH1	scRNA	No	M	2.6	Solid	Yes	No	Yes	No	96	10	20	30	20	A	GTR	No	Yes	Yes	No
SCH2	scRNA	No	F	3.4	Cystic	Yes	No	Yes	Yes	40	100	100	100	100	D	STR	Yes	Yes	No	No
SCH3	scRNA	No	F	2.5	Cystic	Yes	No	No	No	0	100	100	100	100	D	STR	Yes	Yes	No	No
SCH4	scRNA	Yes	F	3.7	Solid	Yes	No	No	Yes	0	75	80	75	76.667	D	STR	Yes	Yes	Yes	Yes
SCH5	scRNA	Yes	M	2	Solid	Yes	No	No	No	4	60	55	85	70	D	GTR	No	Yes	No	No
SCH6	scRNA	Yes	M	3.1	Solid	No	No	Yes	No	96	20	20	25	21.667	A	GTR	No	Yes	Yes	No
SCH7	scRNA	Yes	F	1.3	Solid	Yes	No	Yes	No	40	20	65	60	48.333	D	GTR	No	Yes	No	No
SCH8	scRNA	No	F	1.4	Solid	Prior Tx	Yes	No	No	0	100	100	100	100	D	GTR	No	Yes	No	No
SCH9	scRNA	Yes	M	0.6	Solid	Yes	No	Yes	No	0	100	100	100	100	D	GTR	No	Yes	No	No
SCH13	scRNA	Yes	F	3.6	Cystic	Prior Tx	Yes	No	No	0	100	100	100	100	D	STR	Yes	Yes	No	No
SCH14	scRNA	Yes	F	2.1	Solid	Prior Tx	Yes	No	Yes	0	100	100	100	100	D	STR	Yes	Yes	Yes	No
SCH18	snRNA	No	M	1.9	Solid	Prior Tx	No	No	No	0	100	100	100	100	D	GTR	Yes	Yes	No	No
SCH20	snRNA	No	F	2.1	Cystic	Yes	No	No	No	82	20	15	35	23.333	A	STR	Yes	Yes	No	No
SCH21	snRNA	No	M	3.2	Cystic	Yes	No	Yes	Yes	0	100	100	100	100	D	STR	Yes	Yes	Yes	No
SCH22	snRNA	No	M	3.9	Solid	Yes	No	No	No	45	100	100	100	100	D	STR	Yes	Yes	No	No

Supplementary Table 2. NF2 mutations derived from WES analysis.

ID	Chr	Start	End	Ref	Alt	Gene	ExonicFunc	VAF	tumor_readcounts (ref, alt)	normal_readcounts (ref, alt)
SCH1	chr22	29654719	29654719	A	-	NF2	frameshift deletion	0.83	16,78	63,0
SCH1	chr22	29654720	29654720	A	C	NF2	nonsynonymous SNV	0.821	17,78	63,0
SCH3	chr22	29654670	29654680	ACCCCAGTGTT	-	NF2	frameshift deletion	0.137	170,27	193,0
SCH3	chr22	29671882	29671883	GA	-	NF2	frameshift deletion	0.174	190,40	355,3
SCH4	chr22	29655642	29655649	ACTGCTTG	-	NF2	frameshift deletion	0.124	113,16	155,1
SCH4	chr22	29674841	29674847	AAGAGGC	-	NF2	frameshift deletion	0.061	46,3	89,1
SCH5	chr22	29655678	29655678	T	A	NF2	nan	0.359	25,14	77,0
SCH5	chr22	29678276	29678276	C	-	NF2	frameshift deletion	0.383	29,18	129,0
SCH6	chr22	29636789	29636789	C	A	NF2	stopgain	0.34	198,102	360,0
SCH6	chr22	29654669	29654669	G	-	NF2	frameshift deletion	0.371	78,46	215,0
SCH8	chr22	29654667	29654667	-	CGACCCCAGTGTTCACAAG	NF2	frameshift insertion	0.365	132,76	150,1
SCH8	chr22	29655669	29655669	C	T	NF2	stopgain	0.275	148,56	132,0
SCH13	chr22	29661339	29661339	G	A	NF2	synonymous SNV	0.336	223,113	418,2
SCH14	chr22	29678250	29678250	A	-	NF2	frameshift deletion	0.233	198,60	364,2
SCH18	chr22	29655640	29655640	T	-	NF2	frameshift deletion	0.262	169,60	177,0
SCH18	chr22	29655643	29655643	C	T	NF2	nonsynonymous SNV	0.261	173,61	180,0
SCH20	chr22	29654649	29654663	CTTTCCAGTATGGTG	-	NF2	frameshift deletion	0.283	66,26	127,2
SCH21	chr22	29673473	29673473	G	T	NF2	stopgain	0.417	77,55	403,0
SCH22	chr22	29642263	29642265	CTT	-	NF2	nonframeshift deletion	0.352	35,19	212,1
SCH22	chr22	29642270	29642285	CGCCGTCCAGGCCAAG	-	NF2	frameshift deletion	0.358	34,19	209,0
SCH22	chr22	29671910	29671972	CAGATGAAAGAAGAAGCAACAATGGCCAACGAAGCACTGGTGATTTCTGAGGGGCTGGGGTTC	-	NF2	nonframeshift deletion	0.159	243,46	473,0

Supplementary Table 3. Number of cells of each cell type with/without chromosome 22q loss from all samples predicted to have chr22q loss by inferCNV and/or WES (SCH1, SCH2, SCH13, SCH20).

Cell type	Num. Chr22q Neutral	Num. Chr22q Loss	% Chr22q Loss
Mucosa	0	0	na
Cycling	316	0	0.00%
BC	101	0	0.00%
TC	2577	0	0.00%
NKC	718	0	0.00%
Mast	31	0	0.00%
Myeloid	23485	6	0.03%
PC_VSMC	317	0	0.00%
Endothelial	281	0	0.00%
Fibroblast	1126	0	0.00%
myeSC	0	209	100.00%
nmSC	0	5174	100.00%

Supplementary Table 4. Number of cells of each Schwann cell subtype with/without chromosome 22q loss from all scRNA samples.

Cell subtype	Num. Chr22q Neutral	Num. Chr22q Loss	% Chr22q Loss
Core	1547	711	31.49%
MHC II	887	555	38.49%
Stress	974	381	28.12%
Hypoxia	348	55	13.65%
Myelinating	326	27	7.65%
Repair-like	329	253	43.47%
IFN response	262	37	12.37%
All subtypes	4673	2019	30.17%

Supplementary Table 5. Bulk RNA-seq patient characteristics

ID	Sex	Side	HL	Tin	Vertigo	Size	NF2	Recur	Prior_Sx	Prior_SRS	Postop_SRS	EOR	Postop_Recur
15-04-001	M	Right	Y	N	N	3.17	N	Y	Y	N	N	GTR	N
17-04-006	M	Left	Y	Y	N	4.11	Y	N	N	Y	N	STR	N
17-04-007	F	Left	Y	Y	N	1.55	N	N	N	N	N	GTR	N
19-04-001	F	Left	N	Y	N	2.45	N	N	N	N	N	GTR	N
19-04-002	F	Left	Y	N	N	2.59	N	Y	Y	N	Y	STR	Y
19-04-003	M	Right	Y	N	N	3.74	N	N	N	N	N	GTR	N
19-04-004	F	Left	Y	N	N	3.09	N	N	N	N	N	STR	N
19-04-005	F	Right	Y	Y	Y	2.87	N	N	N	N	N	GTR	N
19-04-007	F	Left	Y	N	N	1.82	N	N	N	N	N	STR	N
19-04-008	F	Right	Y	Y	Y	1.25	N	N	N	N	N	GTR	N
19-04-009	M	Right	Y	N	Y	4.62	N	N	N	N	N	STR	Y
19-04-010	F	Left	Y	Y	N	1.98	N	N	N	N	N	GTR	N
20-04-001	F	Right	Y	N	N	3.41	N	N	N	N	N	GTR	N
20-04-002	M	Left	Y	Y	Y	1.44	N	N	N	N	N	GTR	N
20-04-005	M	Right	Y	Y	Y	1.63	N	N	N	N	N	GTR	N
20-04-008	F	Left	Y	Y	N	1.23	N	N	N	N	N	GTR	N
21-04-001	F	Left	Y	Y	N	2.68	N	N	N	N	N	GTR	N
21-04-002	F	Left	Y	Y	N	1.23	N	N	N	N	N	GTR	N
21-04-003	F	Right	Y	Y	N	2.96	N	N	N	N	N	GTR	N
21-04-004	F	Left	Y	N	Y	1.63	N	N	N	N	N	GTR	N
21-04-005	F	Right	Y	N	N	2.47	N	N	N	N	N	STR	N
21-04-006	M	Left	N	Y	Y	2.85	N	N	N	N	N	GTR	N

Supplementary Table 6. CellBender Parameters

Sample	Expected Cells	Total Droplets	Low Count Threshold	FPR	Epochs	Learning Rate	Z-dims	Z-layers	Model	Training Fraction
SCH1	7000	800000	25	0.01	150	0.000001	100	500	Full	0.9
SCH2	8000	800000	15	0.01	150	0.000002	100	500	Full	0.9
SCH3	2500	800000	10	0.01	150	0.000001	200	1000	Full	0.9
SCH4	5000	800000	25	0.01	150	0.000001	100	500	Full	0.9
SCH5	5000	800000	15	0.01	150	0.000001	100	500	Full	0.9
SCH6	7000	800000	10	0.01	150	0.000001	200	500	Full	0.9
SCH7	4000	800000	10	0.01	150	0.000002	100	500	Full	0.9
SCH8	8000	600000	10	0.01	150	0.000001	100	500	Full	0.9
SCH9	5000	700000	5	0.01	150	0.0000005	150	1000	Full	0.9
SCH13	10000	900000	10	0.01	150	0.000001	100	500	Full	0.9
SCH14	9000	500000	5	0.01	150	0.000002	100	500	Full	0.9
SCH20	18000	750000	10	0.01	150	0.000001	100	500	full	0.9
SCH21	18000	900000	10	0.01	150	0.0000005	100	500	full	0.9
SCH22	20000	900000	10	0.01	150	0.0000005	100	500	full	0.9
