# Supplementary material for: Single-cell multi-omic analysis of the vestibular schwannoma ecosystem uncovers a nerve injury-like state
Source: Nat Commun. 2024 Jan 12;15:478. doi: 10.1038/s41467-023-42762-w (PMC10786875; doi:10.1038/s41467-023-42762-w)
Supplement: Supplementary file 3 — Reporting Summary [file 41467_2023_42762_MOESM3_ESM.pdf]

nature portfolio

Corresponding author(s): Albert H Kim, Sidhart V Puram, Allegra A Petti

Last updated by author(s): Sep 12, 2023

Reporting Summary

Nature Portfolio wishes to improve the reproducibility of the work that we publish. This form provides structure for consistency and transparency in reporting. For further information on Nature Portfolio policies, see our Editorial Policies and the Editorial Policy Checklist.

Statistics

For all statistical analyses, confirm that the following items are present in the figure legend, table legend, main text, or Methods section.

n/a	Confirmed	
☐	☒	The exact sample size (*n*) for each experimental group/condition, given as a discrete number and unit of measurement
☐	☒	A statement on whether measurements were taken from distinct samples or whether the same sample was measured repeatedly
☐	☒	The statistical test(s) used AND whether they are one- or two-sided<br>*Only common tests should be described solely by name; describe more complex techniques in the Methods section.*
☐	☒	A description of all covariates tested
☐	☒	A description of any assumptions or corrections, such as tests of normality and adjustment for multiple comparisons
☐	☒	A full description of the statistical parameters including central tendency (e.g. means) or other basic estimates (e.g. regression coefficient) AND variation (e.g. standard deviation) or associated estimates of uncertainty (e.g. confidence intervals)
☐	☒	For null hypothesis testing, the test statistic (e.g. *F*, *t*, *r*) with confidence intervals, effect sizes, degrees of freedom and *P* value noted<br>*Give P values as exact values whenever suitable.*
☒	☐	For Bayesian analysis, information on the choice of priors and Markov chain Monte Carlo settings
☒	☐	For hierarchical and complex designs, identification of the appropriate level for tests and full reporting of outcomes
☒	☐	Estimates of effect sizes (e.g. Cohen's *d*, Pearson's *r*), indicating how they were calculated

Our web collection on statistics for biologists contains articles on many of the points above.

Software and code

Policy information about availability of computer code

Data collection	scRNA-seq data was aligned using 10x Genomics Cellranger 4.0 for fresh tissue and 6.0 for frozen nuclei samples. scATAC-seq data was aligned using 10x Genomics Cellranger ATAC-1.2.0. RNA-seq data were aligned using STAR version 2.7.2b and gene counts were derived with Picard version 2.6.0. Bioconductor EdgeR (v3.2) used to adjust samples for differences in library size
Data analysis	Data analysis and visualization were performed in R (v4.3.0) using using the following packages: ArchR 1.0.1, Seurat 4.0.0, GEOquery v2.58.0, Biobase v2.50.0, Limma v3.46.0, ComplexHeatmap v2.11.1, Clusterprofiler v3.18.1, QuPath-0.3.1, STAR version 2.7.2b, Picard v2.6.0, CellChat v1.5.0, NMF 0.23.0, Ggpubr 0.4.0, Ggplot2 v3.3.3, InferCNV v1.14.0, circlize v0.4.12, VarTrix v1.1.22, NicheNet v 1.1.1<br>CIBERSORTx was used for deconvolution analysis<br>MACS2 v2.2.7.1 was used for peak calling in ArchR<br>CellBender v0.1.0 was used for ambient RNA correction<br>Scrublet v0.2.1 and ScanPy v1.6.0 were used for doublet detection<br>WES data were analyzed using DRAGEN Bio IT processor using DRAGEN software version 3.10

For manuscripts utilizing custom algorithms or software that are central to the research but not yet described in published literature, software must be made available to editors and reviewers. We strongly encourage code deposition in a community repository (e.g. GitHub). See the Nature Portfolio guidelines for submitting code & software for further information.

Data

Policy information about availability of data

All manuscripts must include a data availability statement. This statement should provide the following information, where applicable:

- Accession codes, unique identifiers, or web links for publicly available datasets
- A description of any restrictions on data availability
- For clinical datasets or third party data, please ensure that the statement adheres to our policy

All scRNA-seq, scATAC-seq, and new bulk RNA-seq data have been made available through the Gene Expression Omnibus with GEO accession GSE216784 without restriction.
All WES data is available through the database of Genotypes and Phenotypes (dbGaP) with accession phs003318.v1.p1.
Raw data from previously published studies were obtained as follows: RNA-seq and expression microarray data that were publicly available were downloaded (GSE39645, GSE141801, GSE108524, EGAS00001001886); data from Aaron et al (Otol Neurotol, 2020) were kindly shared upon request.

Research involving human participants, their data, or biological material

Policy information about studies with human participants or human data. See also policy information about sex, gender (identity/presentation), and sexual orientation and race, ethnicity and racism.

Reporting on sex and gender	Patients were classified by their biological sex. Gender identity was not available for included subjects.
Reporting on race, ethnicity, or other socially relevant groupings	Race, ethnicity, or other socially relevant groupings were not included as they have not been suspected to have an association with vestibular schwannoma.
Population characteristics	Patients were adult (18 years or older) with a diagnosis of sporadic vestibular schwannoma who underwent surgical resection of their tumor.
Recruitment	Patients >18 years of age presenting for surgery were recruited
Ethics oversight	All patients recruited through Washington University provided written informed consent to participate in the study following Institutional Review Board Approval (Protocol #201111001 , #201103136, and #201409046). All patients recruited from Baylor College of Medicine provided written informed consent, and tumor tissues were collected under an institutional review board (IRB)-approved protocol at BCM by the Human Tissue Acquisition and Pathology Core (Protocol H-14435).

Note that full information on the approval of the study protocol must also be provided in the manuscript.

Field-specific reporting

Please select the one below that is the best fit for your research. If you are not sure, read the appropriate sections before making your selection.

[x] Life sciences [ ] Behavioural & social sciences [ ] Ecological, evolutionary & environmental sciences

For a reference copy of the document with all sections, see nature.com/documents/nr-reporting-summary-flat.pdf

Life sciences study design

All studies must disclose on these points even when the disclosure is negative.

Sample size	Sample size for the scRNA-seq scATAC-seq analyses were based on availability of vestibular schwannoma tumor samples, which are a relatively rare clinical entity. Our 15 included samples are similar in size to other similarly exploratory studies of human tumors. Analyses involving previously published datasets did not have a sample size calculation as they were similarly based on availability of data.
Data exclusions	Cells that did not pass QC or were classified as doublets were excluded from further analysis.
Replication	For in vitro experiments, all experiments were repeated three separate times with separate passages of cells to ensure biologic validity of the results. Each experiment had three technical replicates for each condition (n = 3 for both migration and proliferation assays).
Randomization	The impact of treatment was not assessed in patients, and therefore randomization of patients is not relevant to this study.
Blinding	Tissue samples were used in a de-identified manner, and therefore blinding is not relevant to this study.

Reporting for specific materials, systems and methods

We require information from authors about some types of materials, experimental systems and methods used in many studies. Here, indicate whether each material, system or method listed is relevant to your study. If you are not sure if a list item applies to your research, read the appropriate section before selecting a response.

Materials & experimental systems

n/a	Involved in the study	
☐	☒	Antibodies
☐	☒	Eukaryotic cell lines
☒	☐	Palaeontology and archaeology
☒	☐	Animals and other organisms
☒	☐	Clinical data
☒	☐	Dual use research of concern
☒	☐	Plants

Methods

n/a	Involved in the study	
☒	☐	ChIP-seq
☒	☐	Flow cytometry
☒	☐	MRI-based neuroimaging

Antibodies

Antibodies used

Ki67 1:200 dilution (Abcam, clone SP6, catalog # ab16667), CD45 1:200 dilution (Agilent, clone 2B11 + PD7/26, catalogue # M0701), MHC II 1:400 dilution (Cell Signaling Technologies, clone LGII-612.4, catalog # 68258), Ngfr 1:100 dilution (abcam, clone NGFR/1965, catalog # ab224651), S100 1:25 dilution (Invitrogen, clone PA1-26313, catalog # PA1-26313), Sox10 1:100 dilution (Cell Signaling Technology, clone E6B6I, catalog # 69661), SMARCC1 1:800 dilution (Cell Signaling Technology, clone D7F8S, catalog # 11956), CTCF 1:1000 dilution (Cell Signaling Technology, clone D31H2, catalog #3418).

Validation

Antibodies were validated via staining with positive control tissues, per manufacturer recommendations, as follows: Human tonsil tissue for MHC II, Ki67 and CD45; human melanoma tissue for Ngfr, S100 and Sox10; human colon carcinoma for CTCF and SMARCC1

Eukaryotic cell lines

Policy information about cell lines and Sex and Gender in Research

Cell line source(s)

Human Schwann cells (HSC) were shared by Dr. Gelareh Zadeh.

Authentication

The cells were not authenticated.

Mycoplasma contamination

The cells were tested and confirmed to be free of mycoplasma contamination.

Commonly misidentified lines (See ICLAC register)

No commonly misidentified cell lines were used in this study.
