# Supplementary material for: Single-cell multi-omic analysis of the vestibular schwannoma ecosystem uncovers a nerve injury-like state
Source: Nat Commun. 2024 Jan 12;15:478. doi: 10.1038/s41467-023-42762-w (PMC10786875; doi:10.1038/s41467-023-42762-w)
Supplement: Supplementary file 4 — Description of Additional Supplementary Files [file 41467_2023_42762_MOESM4_ESM.pdf]

Description of Additional Supplementary Files

Supplementary Data 1
Description: Differential gene expression of cell types in the VS TME

Supplementary Data 2
Description: Gene signatures for cell types identified in studies of murine peripheral nerves

Supplementary Data 3
Description: Somatic variants detected using whole exome sequencing.

Supplementary Data 4
Description: InferCNV HMMI3 region data

Supplementary Data 5
Description: Passing filter copy number altered segments from whole exome sequencing data.

Supplementary Data 6
Description: DEGs of Schwann cell subtypes

Supplementary Data 7
Description: GO BP enrichment among VS-SC subpopulations

Supplementary Data 8
Description: DEGs of stromal cell subpopulations

Supplementary Data 9
Description: DEGs of NK and T-cell subpopulations

Supplementary Data 10
Description: Myeloid cell metaprogram genes

Supplementary Data 11
Description: Imputed Cibersortx cell type fractions

Supplementary Data 12
Description: CellChat Ligand-Receptor Predictions

Supplementary Data 13
Description: Probes added to IDT IDT Exome Hyb Panel v2 for whole exome sequencing.

Supplementary Data 14
Description: Sequencing QC Data

Supplementary Data 15
Description: Single Cell ATAC Sequencing Cell Metadata

Supplementary Data 16
Description: Single Cell RNA Sequencing Cell Metadata
